# Construction of a predictive model for immunotherapy efficacy in lung squamous cell carcinoma based on the degree of tumor-infiltrating immune cells and molecular typing

**DOI:** 10.1186/s12967-022-03565-7

**Published:** 2022-08-12

**Authors:** Lingge Yang, Shuli Wei, Jingnan Zhang, Qiongjie Hu, Wansong Hu, Mengqing Cao, Long Zhang, Yongfang Wang, Pingli Wang, Kai Wang

**Affiliations:** 1 Department of Respiratory and Critical Care Medicine, The Fourth Affiliated Hospital, International Institutes of Medicine, Zhejiang University School of Medicine, Yiwu, China; 2 Department of Respiratory and Critical Care Medicine, The Second Affiliated Hospital of Zhejiang University School of Medicine, Hangzhou, China; 3 Department of Heart Center, The Fourth Affiliated Hospital, International Institutes of Medicine, Zhejiang University School of Medicine, Yiwu, China

**Keywords:** Lung squamous cell carcinoma (LUSC), Tumor-infiltrating immune cells, Immune checkpoint inhibitors, Immunotherapy efficacy, Predictive model, Molecular typing

## Abstract

**Background:**

To construct a predictive model of immunotherapy efficacy for patients with lung squamous cell carcinoma (LUSC) based on the degree of tumor-infiltrating immune cells (TIIC) in the tumor microenvironment (TME).

**Methods:**

The data of 501 patients with LUSC in the TCGA database were used as a training set, and grouped using non-negative matrix factorization (NMF) based on the degree of TIIC assessed by single-sample gene set enrichment analysis (GSEA). Two data sets (GSE126044 and GSE135222) were used as validation sets. Genes screened for modeling by least absolute shrinkage and selection operator (LASSO) regression and used to construct a model based on immunophenotyping score (IPTS). RNA extraction and qPCR were performed to validate the prognostic value of IPTS in our independent LUSC cohort. The receiver operating characteristic (ROC) curve was constructed to determine the predictive value of the immune efficacy. Kaplan–Meier survival curve analysis was performed to evaluate the prognostic predictive ability. Correlation analysis and enrichment analysis were used to explore the potential mechanism of IPTS molecular typing involved in predicting the immunotherapy efficacy for patients with LUSC.

**Results:**

The training set was divided into a low immune cell infiltration type (C1) and a high immune cell infiltration type (C2) by NMF typing, and the IPTS molecular typing based on the 17-gene model could replace the results of the NMF typing. The area under the ROC curve (AUC) was 0.82. In both validation sets, the IPTS of patients who responded to immunotherapy were significantly higher than those who did not respond to immunotherapy (*P* = 0.0032 and *P* = 0.0451), whereas the AUC was 0.95 (95% CI = 1.00–0.84) and 0.77 (95% CI = 0.58–0.96), respectively. In our independent cohort, we validated its ability to predict the response to cancer immunotherapy, for the AUC was 0.88 (95% CI = 1.00–0.66). GSEA suggested that the high IPTS group was mainly involved in immune-related signaling pathways.

**Conclusions:**

IPTS molecular typing based on the degree of TIIC in the TME could well predict the efficacy of immunotherapy in patients with LUSC with a certain prognostic value.

**Supplementary Information:**

The online version contains supplementary material available at 10.1186/s12967-022-03565-7.

## Background

Lung cancer is a common malignant tumor worldwide. According to the 2020 global cancer statistics, the mortality and incidence rates of lung cancer rank first and second, respectively [1]. Lung squamous cell carcinoma (LUSC) is the second most common histological subtype of lung cancer with ~ 30% of all cases [2]. Due to the insidious onset and low early diagnosis rate, many patients with LUSC have already passed the opportunity for surgery by the time of diagnosis [3]. The 5-year survival rate of patients with LUSC who receive surgery is still low at 12.4% [4]. Compared with lung adenocarcinoma, LUSC has a low rearrangement rate of *EGFR* gene mutation and *ALK* fusion gene, and strong tumor heterogeneity [5], Therefore, LUSC is limited in gene mutation-based targeted therapy applications [6, 7]. Other treatments such as chemotherapy and radiotherapy also have a limited impact on the long-term survival of patients with LUSC [8]. Thus, patients with LUSC generally have a poor prognosis [9].

In clinical application, immunotherapy plays an integral anti-tumor role by activating the immune system and is rapidly becoming an important tool for cancer treatment. The most widely used immunotherapy is immune checkpoint inhibitors (ICIs), and they have shown promising therapeutic outcomes in non-small cell lung cancer (NSCLC) [10]. However, the response rate of immunotherapy is relatively low, and only a subset of patients show meaningful clinical response or benefit [11]. As a target of PD-1/PD-L1 antibodies, the PD-L1 level in cancer cells as measured by immunohistochemistry is the only FDA-approved and widely used biomarker for predicting response to ICIs in clinical practice. However, the predictive ability of the PD-L1 level is limited, and despite a high PD-L1 level, a proportion of patients receiving ICIs still do not respond; similarly, a negative PD-L1 level also does not reliably preclude a response to PD-1/PD-L1 blockade [12], suggesting there is an urgent need for effective biomarkers capable of screening patients with LUSC according to their likelihood of benefiting from ICI therapy. Beyond the intrinsic factors of tumor cells, studies have identified the tumor microenvironment (TME) characteristics also determine the ICI tumor response [13]. Among them, immune cells play key roles in mediating immune surveillance and regulating tumor growth [14]. Therefore, tumor-infiltrating immune cells (TIICs) may be a potential biomarker to predict the efficacy of immunotherapy.

A clinical prediction model is a tool that combines multiple predictors to evaluate the probability of an individual presenting with a certain disease or clinical outcome. Some clinical prediction models have potential value for screening, diagnosis, treatment, and prognostic prediction of lung cancer [15–17]. With the rapid development of high-throughput sequencing and bioinformatics analysis methods, obtaining cancer-related genomes, transcriptomes, and immune-related information has become readily easier. This has enabled the construction of lung cancer prediction models based on gene-related predictors, which are now widely used in clinical practice.

At present, there is a relative lack of predictive models for the efficacy of immunotherapy in LUSC based on TIIC. Our study intends to construct a predictive model for the efficacy of immunotherapy for patients with LUSC based on the degree of TIIC. First, non-negative matrix factorization (NMF) [18] was used to classify the gene expression profile of patients with LUSC from The Cancer Genome Atlas (TCGA) database. Then, after intersecting differentially expressed genes (DEGs) between NMF typing, survival-related genes, and their comparison with two validation gene sets of patients receiving immunotherapy, a least absolute shrinkage and selection operator (LASSO) analysis was performed [19]. Finally, 17 genes were screened out and the corresponding regression coefficients were obtained, which were used to construct an immunophenotyping score (IPTS) molecular typing, and used to analyze the predictive value of IPTS on the efficacy of immunotherapy for patients with LUSC.

## Method

### Data collection and processing

The clinical information and gene expression profile matrix of patients with LUSC were downloaded from the TCGA database (https://cancergenome.nih.gov, access date: October 15, 2021). A total of 501 samples with complete clinical information and expression profile matrix were selected as the training set to construct the immune efficacy prediction model. Then, the gff3 file (v37, released on October 14, 2021) was downloaded from GENCODE (https://www.gencodegenes.org/human/) [20], the Gene Symbol and ENSG_ID extracted using R v4.1.2, and matched with the TCGA-LUSC expression profile matrix to convert ENSG_ID to Gene Symbol. Next, the count data were transformed into transcripts per kilobase million (TPM) data based on gene length for subsequent analyses.

In addition, the clinical information and gene expression matrix of two data sets of NSCLC with immunotherapeutic efficacy, GSE126044 [21] and GSE135222 [22, 23], were downloaded from the gene expression omnibus (GEO) (https://www.ncbi.nlm.nih.gov/geo, access date: February 11, 2022) database as the validation sets. The GSE126044 dataset was sequenced using the HiSeq 2500 (GPL16791; Illumina, San Diego, CA, USA) platform, with a total of 16 NSCLC samples. The GSE135222 dataset was also sequenced using the GPL16791 platform, with a total of 27 samples, and this dataset contained the prognosis information of progression-free survival (PFS).

### Single-sample gene set enrichment analysis (ssGSEA) to assess the degree of immune cells infiltration

According to the gene signatures of 28 types of immune cells reported by Jia Q [24], we used the “GSVA” package (v1.42.0) [25] and the ssGSEA method to obtain the enrichment scores of 28 types of immune cells in each of the 501 LUSC cases in the training set. These 28 kinds of immune cells can be divided into cell types executing anti-tumor immunity (including activated CD4 T, activated CD8 T, activated dendritic, CD56 bright natural killer (NK), central memory CD4 T, central memory CD8 T, effector memory CD4 T, effector memory CD8 T, NK, NK T, type 1 T helper, and type 17 T helper cells), cell types executing pro-tumor, immune suppressive function (including CD56 dim NK, immature dendritic, myeloid-derived suppressor, plasmacytoid dendritic, regulatory T, type 2 T helper cells, neutrophils, and macrophages), and other cell types (activated B, gamma delta T, immature B, mast, memory B, T follicular helper cells, eosinophils, and monocytes).

### NMF typing

After normalizing the above matrix of immune cell enrichment scores, we used the “NMF” package (v0.23.0) [26] for typing with *rank* set to 2:10, the *method* to brunet, and *nrun* to 100. Then, we used the “Rtsne” package (v0.15) [27] and the “prcomp” function of the “stats” package (v3.6.0) [28] to perform dimensionality reduction analysis to verify the feasibility of the NMF typing results. In addition, to clarify the differences in TIIC between different types, we performed correlation analysis and difference analysis on the anti-tumor immunity enrichment scores and the pro-tumor immunity enrichment score between different subtypes, and analyzed the difference in the enrichment scores of 28 kinds of TIIC respectively, to further demonstrate the reliability of NFM typing results.

### DEGs of NMF typing and their functional enrichment analysis

To clarify the DEGs of different NMF types, we used the “limma” package (v3.50.1) [29] to analyze the DEG profile, and used the “p.adjust” function to calculate the significant false discovery rate (FDR, *q*-value) of each gene. FDR (*q*-value) < 0.05 was considered to be statistically significant. Then, the “clusterProfiler” package (v3.14.3) [30] was used for functional enrichment analysis of gene ontology (GO) and Kyoto encyclopedia of genes and genomes (KEGG) to obtain the results of gene set enrichment. The minimum gene set was 5 and the maximum gene set was 5,000, with *P* < 0.05 and FDR < 0.05 considered meaningful. The results were ranked by FDR and the top ten functional enrichment results were plotted.

### Survival analysis and screening for genes affecting overall survival (OS) and disease-free survival (DFS)

To determine whether there is a difference in survival between patients with different molecular types, we grouped the patients according to the NMF types, and then used the “survival” package (v3.3-1) [31] for survival analysis, with the optimal cutoff value calculated using the “surv_cutpoint” function. Then, the “survminer” package (v0.4.9) was used for plotting survival curves [32]. In addition, to screen the genes that have an impact on the prognosis and survival of LUSC in the TCGA database, we first removed the samples with a survival time of fewer than 30 days, and then performed survival analyses on all genes in the gene expression profile to obtain the genes affecting OS and DFS, respectively.

### Construction of an immune efficacy prediction model

Due to the different sequencing platforms or sequencing depths, many genes detected in the training set were not detected in the validation sets. To better use the validation set for verification, we used DEGs, genes affecting the OS and DFS of patients, and genes measured in the two validation sets of GSE126044 and GSE135222. After intersecting these five gene sets, the screened genes were used to construct the immune efficacy prediction model. A Venn diagram was plotted using the online tool Bioinformatics & Evolutionary Genomics (http://bioinformatics.psb.ugent.be/webtools/Venn).

The gene expression profile matrix screened above for modeling and NMF typing were extracted and then the LASSO regression model was constructed using the “glmnet” package (v4.1–3) [33, 34], with *nfold* set to 10 and *λ* equaling lambda.min. After obtaining the regression coefficients of the screened genes, the IPTS equation was constructed based on these coefficients. Then, nomograms and calibration curves were plotted using the “rms” package (v6.2-0) [35] to visualize the regression analysis results. In addition, we plotted a Sankey diagram using the “networkD3” package (v0.4) [36] to visualize the typing results and their corresponding gene signatures.

### Our independent LUSC validation set collection and follow-up

All paraffin sections from 10 cases of LUSC tissues were collected in The Second Affiliated Hospital of Zhejiang University School of Medicine from November 2019 to February 2022. Clinicopathological characteristics and prognostic survival information of these LUSC patients, including ages, gender, TNM stage, clinical stage, tumor size before and after treatment, tumor site, best response evaluation and PD-L1 immunohistochemistry data were acquired. The follow-up date was ended at July 7, 2022, and outpatient and telephone follow-up were performed. This study was approved by the institutional review committee of The Second Affiliated Hospital of Zhejiang University School of Medicine (Approval Number: 2022-0548/I2022685). All the patients have written informed consent before surgery.

### RNA extraction and real-time quantitative PCR (qPCR)

For real-time qPCR analysis, the BIOG RNA FFPE Tissue Kit (Baidai, Changzhou, China) was used to extract total RNA from 10 samples of Paraffin section of lung from patients receiving immunotherapy according to the manufacturer’s instructions. cDNA was synthesized using the HiScript III All-in-one RT SuperMix Perfect (Vazyme, Nanjing, China). Real-time q-PCR was performed to detect the expression of the screened genes using TB Green Premix Ex Taq II (Takara, Dalian, China) to calculate IPTS. Gene expression levels were normalized to the “housekeeping” gene GAPDH. The primers and their sequences were listed in Additional file 2: Table S1.

### Reliability and verification of model for immunotherapy efficacy prediction

To verify the discrimination of NMF typing by the prediction model and whether it can replace the sample typing results, we first calculated the IPTS value of each sample in the training and two validation sets as well as our independent cohort according to both the gene expression value and the constructed IPTS equation. Then, the IPTS and NMF typing results of the training set were integrated, and the receiver operating characteristic (ROC) curves were constructed through the “pROC” package (v1.18.0) [37] to evaluate the ability of the prediction model to judge the NMF typing. Next, according to the cutoff value of the ROC curve, the training set was divided into high and low score groups. The differences and correlations between the high and low IPTSes for different NMF typing, as well as the pro- and anti-tumor immunity enrichment scores between the high and low score groups, were analyzed, respectively. In addition, the differences between the enrichment scores of 28 types of immune cells were also analyzed to clarify the degree of coincidence between the IPTS molecular typing results and the NMF typing results, i.e., whether the substitution of the IPTS molecular typing results for the NMF typing results is reasonable. Besides, since the genes in the model have an impact on the OS and DFS of the training set, we also performed survival analysis and plotted the survival curve to evaluate the prognostic predictive value of the model.

According to the clinical information of patients with LUSC in the TCGA database, almost no patients received immunotherapy. To evaluate whether the constructed immune typing model can predict immunotherapy efficacy, we validated the immunotherapy efficacy in the two validation sets for patients with NSCLC as well as our independent LUSC cohort. First, we compared whether there were significant differences in the IPTSes between groups of patients with lung cancer and different immune responses. Due to the small sample size in the GSE126044 dataset and the IPTS in this dataset with non-normally distributed and uneven variance, the Mann–Whitney rank-sum test was used for the differences between groups in this dataset, and *P* < 0.05 was considered to be statistically significant. And due to the power distribution of IPTSes in our independent LUSC cohort, log2 transformation was conducted before using the student’s t-test. Then, the ROC curve was constructed according to the IPTS and immune response results to evaluate the predictive value of the immune efficacy of the model. In addition, since the GSE135222 dataset and our cohort contain data on the PFS of patients, we further conducted survival analyses to verify the predictive value of immune efficacy and evaluate the prognostic prediction ability of this model.

### Efficacy prediction of other anti-tumor drugs

Genomics of drug sensitivity in cancer (GDSC; https://www.cancerrxgene.org, access date: February 27, 2022) [38] contains the sequencing data of more than 1000 human tumor cell lines and the treatment results of tumor cells by more than 100 anti-tumor drugs, which facilitate finding molecular characteristics of tumors and predicting the response of targets to anti-tumor drugs. The sequencing results of all cell lines in the database and the 50% inhibitory concentration (IC_50_) of cell lines treated with anti-tumor drugs were downloaded, and the results of all 15 cell lines of LUSC sequenced in this database were extracted. Next, the IPTS of the 15 cell lines were calculated and then divided into two groups: 8 cases with high IPTS and 7 cases with low IPTS. The differences in IC_50_ of anti-tumor drugs between the two groups were tested, and the anti-tumor drugs with statistical significance (*P* < 0.05) were selected.

### Analysis of differences and correlation between two IPTS groups in immune microenvironment score and immune molecular typing

Through the “estimate” package (v1.0.13) [39], we evaluated the three immune microenvironment related scores of 501 samples in the training set as well as analyzed the differences and correlations between the high and low IPTS groups. Meanwhile, according to the summary of genotype and immunophenotype by Charoentong [40] and Hu [41], we obtained the following five genetic markers of immune molecular typing, namely chemokines, receptors, major histocompatibility complex (MHC) molecules, immuno-inhibitors, and immuno-stimulators. Next, the enrichment scores of the above five immune molecular typing in the training set were calculated by ssGSEA, and the differences between the high and low score groups were then analyzed. As the current clinically used ICIs are mainly anti-CTLA-4 and anti-PD-1/PD-L1 antibodies, we analyzed the differences and correlations between the expression of four immune checkpoints CTLA-4, PD-1 (PDCD1), and its two ligands PD-L1 (CD274) and PDL-2 (PDCD1LG2) in the training set between high and low score groups.

### Gene set enrichment analysis (GSEA)

We performed GSEA (https://www.gsea-msigdb.org/gsea, access date: March 1, 2022) [42] on the gene expression profile of the training set based on the high and low IPTS groups. First, the subset of c2.cp.kegg.v7.4.symbols.gmt were downloaded to assess relevant pathways and molecular mechanisms. Based on the gene expression profile and IPTS grouping, the minimum gene set was 5, the maximum gene set was 5000, and the number of resampling was set to 1000. Then, ranked by the normalized enrichment score (NES), the top seven results were visualized. A normalized *P*-value (NP) < 0.05 was considered to be meaningful.

### Statistical analysis

All data in this study were analyzed and plotted using R v4.1.2 and Prism v8.0.1 (GraphPad, San Diego, CA, USA). The parameters not mentioned in the methods were all default parameters, and the data visualization not mentioned was all plotted by the “ggplot2” package (v3.3.5) [43]. Continuous variables were displayed as mean ± standard deviation. Unless mentioned otherwise mentioned, the student’s t-test was used to compare the differences between the two groups. The differences between groups of discrete variables were analyzed using the chi-squared test. The Pearson test was used for correlation analysis, and the log-rank test was used for survival analysis. *P* < 0.05 was considered as a statistically significant difference (*P < 0.05, **P < 0.01, ***P < 0.001, ****P < 0.0001).

## Results

### NMF typing divides the training set into low and high immune cell infiltration types

**Fig. 1:**
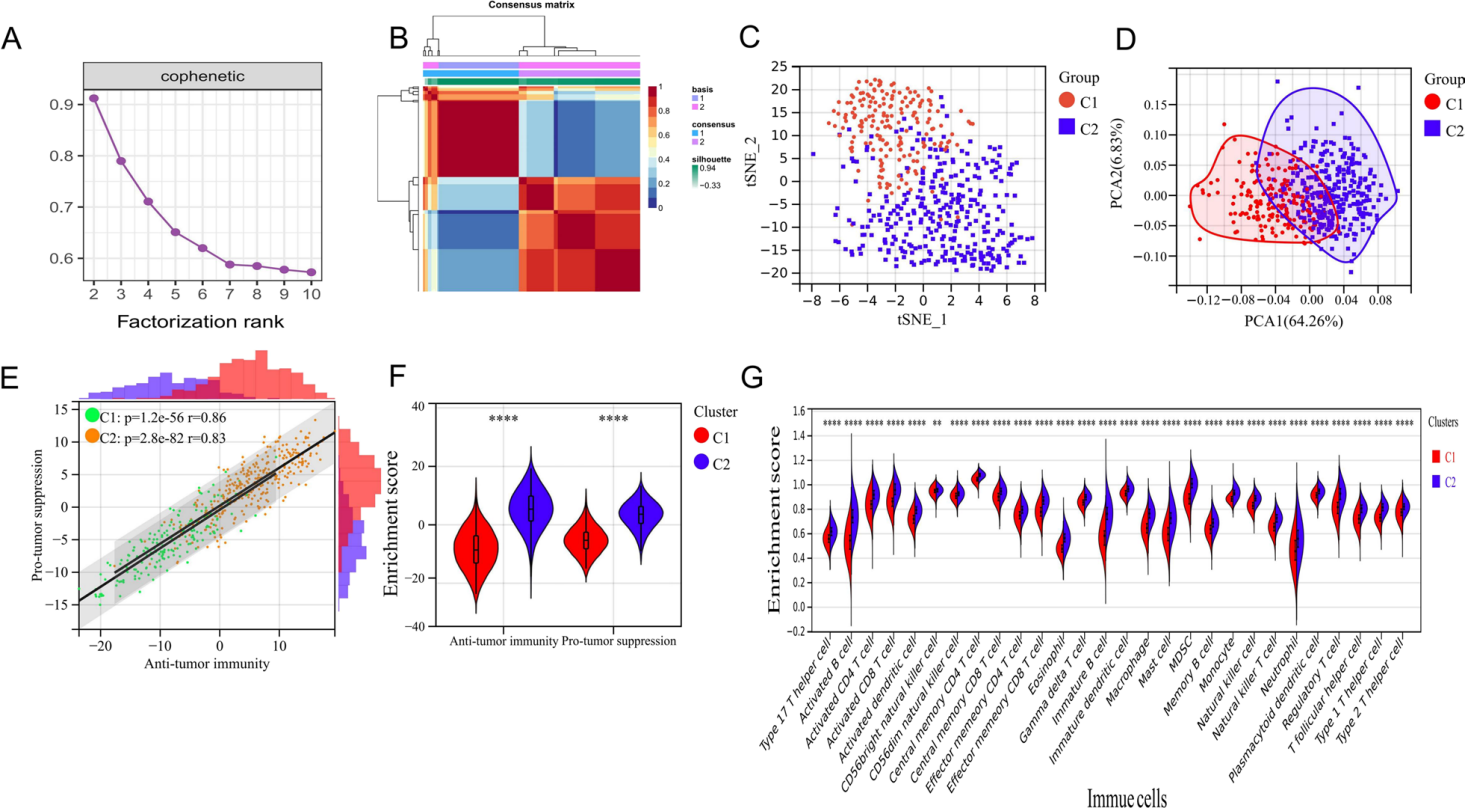
NMF typing divides the training set into low and high immune cell infiltration types. **A** NMF typing using the enrichment score matrix of 28 types of immune cells in the training set. Cophenetic correlation coefficient *k* = 2–10; **B** Heat map of the samples typing in the training set for NMF typing = 2; **C** t-SNE analysis divides patients with LUSC in the training set into two subtypes; **D** PCA divides patients with LUSC in training set into two subtypes; **E** correlation analysis between the enrichment scores of anti- and pro-tumor immunity in two NMF types; **F** enrichment score of anti- and pro-tumor immunity between C2 and C1 patients; **G** enrichment scores of 28 types of immune cells between types C1 and C2 patients. **P < 0.01; ****P < 0.0001

The clinical information of the training, two validation sets and our independent LUSC cohort is detailed in Additional file 3: Table S2. NMF was used to classify 501 patients with LUSC in the training set. It was found that when the rank value was 2–3, the cophenetic typing index decreased the most (Fig. 1A). Therefore, a rank value of 2 was selected, and the patients were divided into a low immune cell infiltration type (cluster 1; C1) and a high immune cell infiltration type (cluster 2; C2). The typing efficacy of other NMF indicators is shown in Additional file 1: Figure S1. The heat map also showed that when the number of types was limited to two, the samples of the training set could be well distinguished (Fig. 1B). The discriminatory capacity when more subtypes were used in the training can be seen in Additional file 1: Fig. S2. In addition, dimensionality reduction by t-distributed stochastic neighbor embedding (tSNE) (Fig. 1C) and principal component analysis (PCA) (Fig. 1D) showed that C1 and C2 had good discrimination ability, suggesting the feasibility of using this classification.

There were significant positive correlations between the scores of cell types executing anti-tumor immunity and those executing pro-tumor immunity in both the C1 and C2 patients (Fig. 1E), with a correlation coefficient of 0.86 in C1 patients (*P* = 1.2e−56) and 0.83 in C2 patients (*P* = 2.8e−82). The enrichment score of the anti- (*P* < 0.0001) and pro-tumor immunity (*P* < 0.0001) were significantly higher in C2 patients than in C1 patients (Fig. 1F). In addition, there were significant differences in the enrichment scores of 28 types of immune cells, with significantly lower scores in type C1 than type C2 (Fig. 1G). It is suggested that type C1 of LUSC can be regarded as a “cold tumor” with low infiltration of immune cells, while type C2 tends to be a “hot tumor”, i.e., a tumor with high infiltration of immune cells. These results reveal that NMF typing had a strong ability to distinguish the degree of immune cell infiltration in the training set.

### Clinicopathological characteristics between NMF types

**Fig. 2:**
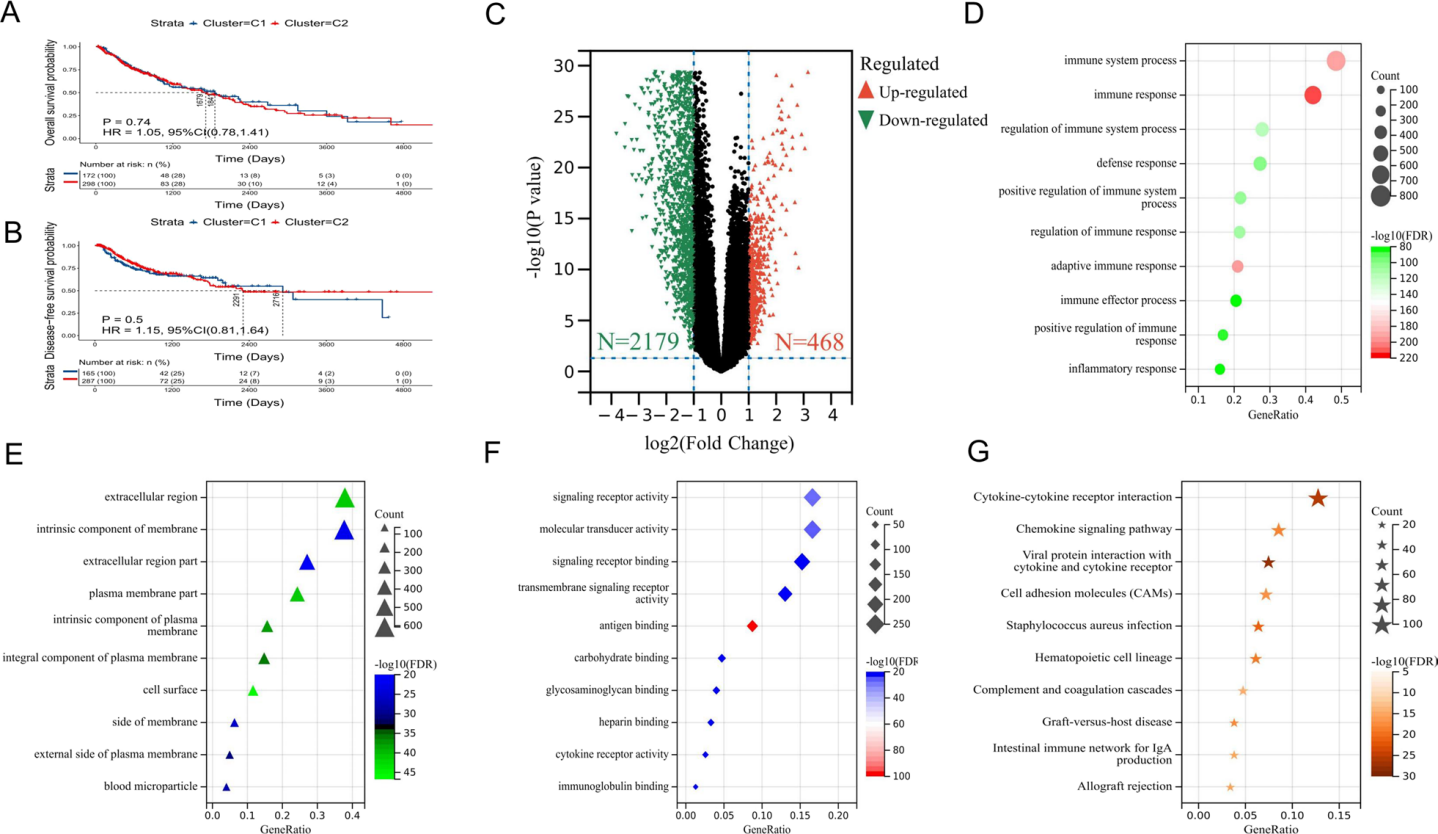
Differentially expressed genes (DEGs) between NMF types are mainly involved in immune system regulation. **A** Survival analysis shows no significant difference in OS between NMF types; **B** survival analysis shows no significant difference in DFS between NMF types; **C** significant DEGs between NMF types: 468 up-regulated and 2179 down-regulated in type C1 compared with type C2; **D**–**G** top ten enriched **D** biological processes, **E** cellular components, **F** molecular functions, and **G** KEGG pathways in DEGs

According to the NMF typing, we performed difference analyses on several important clinicopathological characteristics of the patients in the training set, including age, gender, TNM stage, and clinical stage. The results only showed significant differences in gender between the two subtypes. A total of 36 patients in type C1 were female (7.19%), which was significantly less than the 94 patients (18.76%) in type C2 (*P* = 0.0112). There were no differences in other clinicopathological characteristics such as age and stage between the subtypes (Table 1). Subsequent survival analysis revealed no significant difference between the two subtypes for either the OS (*P* = 0.74, Fig. 2A) or DFS (*P* = 0.5, Fig. 2B) for patients with LUSC.

**Table 1:** Correlation between clinicopathological characteristics and NMF typing in the TCGA database

Characteristics	C1 (*N* = 185)	C2 (*N* = 316)	Total (*N* = 501)	*P*-value
Age				
Mean ± standard deviation	66.33 ± 8.74	67.71 ± 8.46	67.20 ± 8.58	0.0836
Median [min, max]	68 [39, 85]	68 [40, 90]	68 [39, 90]	
Gender				
Female	36 (7.19%)	94 (18.76%)	130 (25.95%)	0.0112*
Male	149 (29.74%)	222 (44.31%)	371 (74.05%)	
T				
T1	36 (7.19%)	78 (15.57%)	114 (22.75%)	0.5468
T2	112 (22.36%)	181 (36.13%)	293 (58.48%)	
T3	29 (5.79%)	42 (8.38%)	71 (14.17%)	
T4	8 (1.60%)	15 (2.99%)	23 (4.59%)	
N				
N0	112 (22.36%)	207 (41.32%)	319 (63.67%)	0.1161
N1	57 (11.38%)	74 (14.77%)	131 (26.15%)	
N2	16 (3.19%)	24 (4.79%)	40 (7.98%)	
N3	0	5 (1.00%)	5 (1.00%)	
N_x_	0	6 (1.20%)	6 (1.20%)	
M				
M0	149 (29.74%)	262 (52.30%)	411 (82.04%)	0.4213
M1	1 (0.20%)	6 (1.20%)	7 (1.40%)	
M_x_	34 (6.79%)	45 (8.97%)	79 (15.76%)	
Unknown	1 (0.20%)	3 (0.60%)	4 (0.80%)	
Stage				
Stage I	80 (15.97%)	164 (32.73%)	244 (48.70%)	0.1160
Stage II	68 (13.57%)	94 (18.76%)	162 (32.34%)	
Stage III	35 (6.99%)	49 (9.78%)	84 (16.77%)	
Stage IV	1 (0.20%)	6 (1.20%)	7 (1.40%)	
Unknown	1 (0.20%)	3 (0.60%)	4 (0.80%)	
IPTS groups				
High score	52 (10.38%)	254 (50.70%)	306 (61.08%)	< 0.0001****
Low score	133 (26.55%)	62 (12.38%)	195 (38.92%)	

Bold values indicate *P* < 0.05 and *P < 0.05, ****P < 0.0001

### DEGs between NMF types are mainly involved in immune system regulation

Through differential analysis, 468 genes were significantly up-regulated in type C1 compared with type C2, and 2179 genes were significantly down-regulated (Fig. 2C). GO enrichment analysis showed that DEGs were mainly enriched in the following biological processes: immune system process (48.50%), immune response (42.02%), regulation of immune system process (27.84%), etc. (Fig. 2D); In terms of cellular components, they were mainly enriched in the extracellular region (37.87%), intrinsic components of the membrane (15.40%), and integral components of the membrane (14.78%), etc. (Fig. 2E); while in terms of molecular functions, these genes were mainly enriched in signaling receptor activity (16.61%), molecular transducer activity (16.61%), and signaling receptor binding (15.25%), etc. (Fig. 2F). KEGG enrichment analysis showed that DEGs were mainly involved in cytokine-cytokine receptor interaction (12.75%), chemokine signaling pathway (8.55%), viral proteins, viral protein interaction with cytokine and cytokine receptor (7.46%), and other pathways (Fig. 2G). Additional file 4: Table S3 shows all meaningful GO and KEGG enrichment analysis results.

### Construction of an immune infiltration prediction model based on 17 genes

**Fig. 3:**
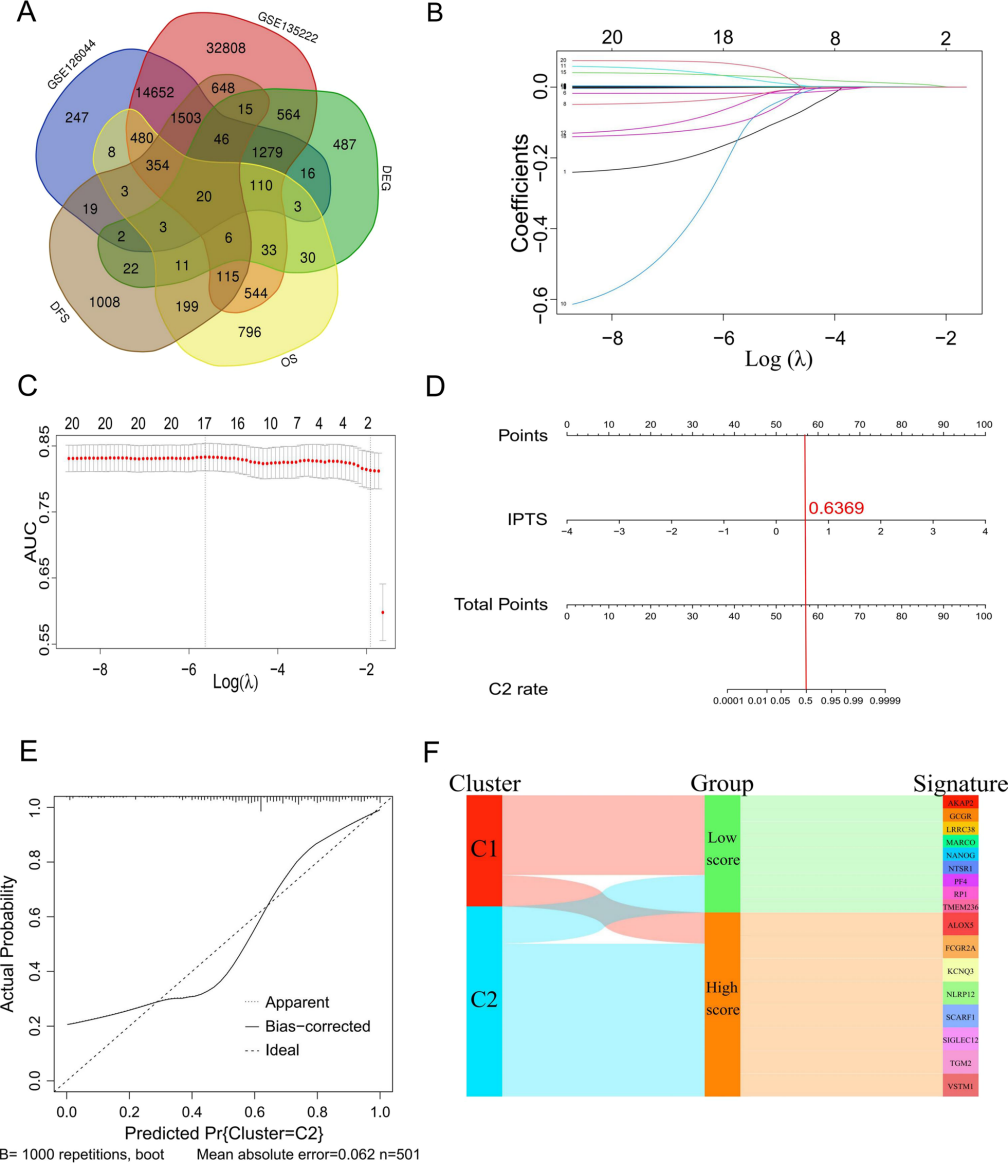
Construction of an immune infiltration prediction model based on 17 genes. **A** Venn diagram of the intersection of differentially expressed genes, genes affecting overall survival and disease-free survival of patients in the training set, as well as in the two validation sets GSE126044 and GSE135222; **B** Lasso coefficient distribution diagram of 20 genes with x-coordinate log (λ) for screening the best tuning parameter (λ); **C** screening of the tuning parameter in the lasso regression model based on tenfold cross-validation; Plotting was performed based on this value and the AUC value of the ROC curve. A vertical dashed line was drawn at the best value by using the minimum standard and 1 standard error of the minimum standard (1-SE standard); **D** nomogram plotted based on the IPTS. IPTS could predict whether the patient had an NMF type of either C1 or C2. The higher the IPTS, the higher the probability of the patient having type C2. When IPTS = 0.6369, the probability of the patient in the C1 and C2 types was 50%. **E** Calibration curve plot according to lasso regression analysis. The x-coordinate represented the probability that the model predicts type C2 for patients, and the y-ordinate represented the actual probability. **F** Sankey diagram plotted according to NMF or IPTS subtyping, and 17 gene signatures. The low IPTS group tended towards type C1, while the high IPTS group was more representative of type C2

Although NMF typing could better distinguish the abundance of TIICs, it could not predict the survival prognosis of patients. Therefore, a new predictive model needed to be developed on this premise. A total of 20 genes were obtained for constructing the prediction model, by taking the intersection of the following five gene sets: DEGs, genes affecting the OS and DFS of patients, and genes sequenced in the two validation sets (Fig. 3A). Through LASSO regression, a total of 17 genes with a regression coefficient were selected (Fig. 3B). In addition, it can be seen from Fig. 3C that the model had a higher ROC area under the curve (AUC) value when considering the minimum value of the tuning parameter (*λ*).

Based on these 17 genes and their regression coefficients, the IPTS model was constructed as follows: IPTS = 0.4869250211 − 0.1428834537 × *AKAP2* expression value − 0.12060842 × *NANOG* expression value – 0.0951070744 × *TMEM236* expression value − 0.0436119966 × *NTSR1* expression value − 0.0258542814 × *LRRC38* expression value − 0.0170225681 × *GCGR* expression value − 0.0011330363 × *MARCO* expression value − 0.0008511336 × *PF4* expression value − 0.0004418332 × *RP1* expression value + 0.0023249088 × *ALOX5* expression value + 0.0021763779 × *FCGR2A* expression value + 0.0006362408 × *KCNQ3* expression value + 0.0247048306 × *NLRP12* expression value + 0.0314720069 × *SCARF1* expression value + 0.0013954206 × *SIGLEC12* expression value + 0.0004957628 × *TGM2* expression value + 0.0617891897 × *VSTM1* expression value.

In addition, the predictive effect of the prognostic model on type C2 was visualized by constructing a nomogram (Fig. 3D). It can be seen that when IPTS = 0.6369, the probability of patients in type C1 and C2 was 50%, and the probability of patients in type C2 was higher when the value exceeded 0.6369. Besides, under the condition of 1000 repetitions, the mean absolute error (MAE) of the calibration curve (Fig. 3E) was 0.062, and the curve fitting was suitable, indicating a sound prediction effect. As can be seen from this model, there were nine genes with regression coefficient < 0—which can be used as gene signatures of type C1; and eight genes with regression coefficient > 0—which can be used as gene signatures of type C2 (Fig. 3F). Thus, molecular typing was achieved to a certain extent.

### Molecular typing based on IPTS prediction model can replace NMF typing

**Fig. 4:**
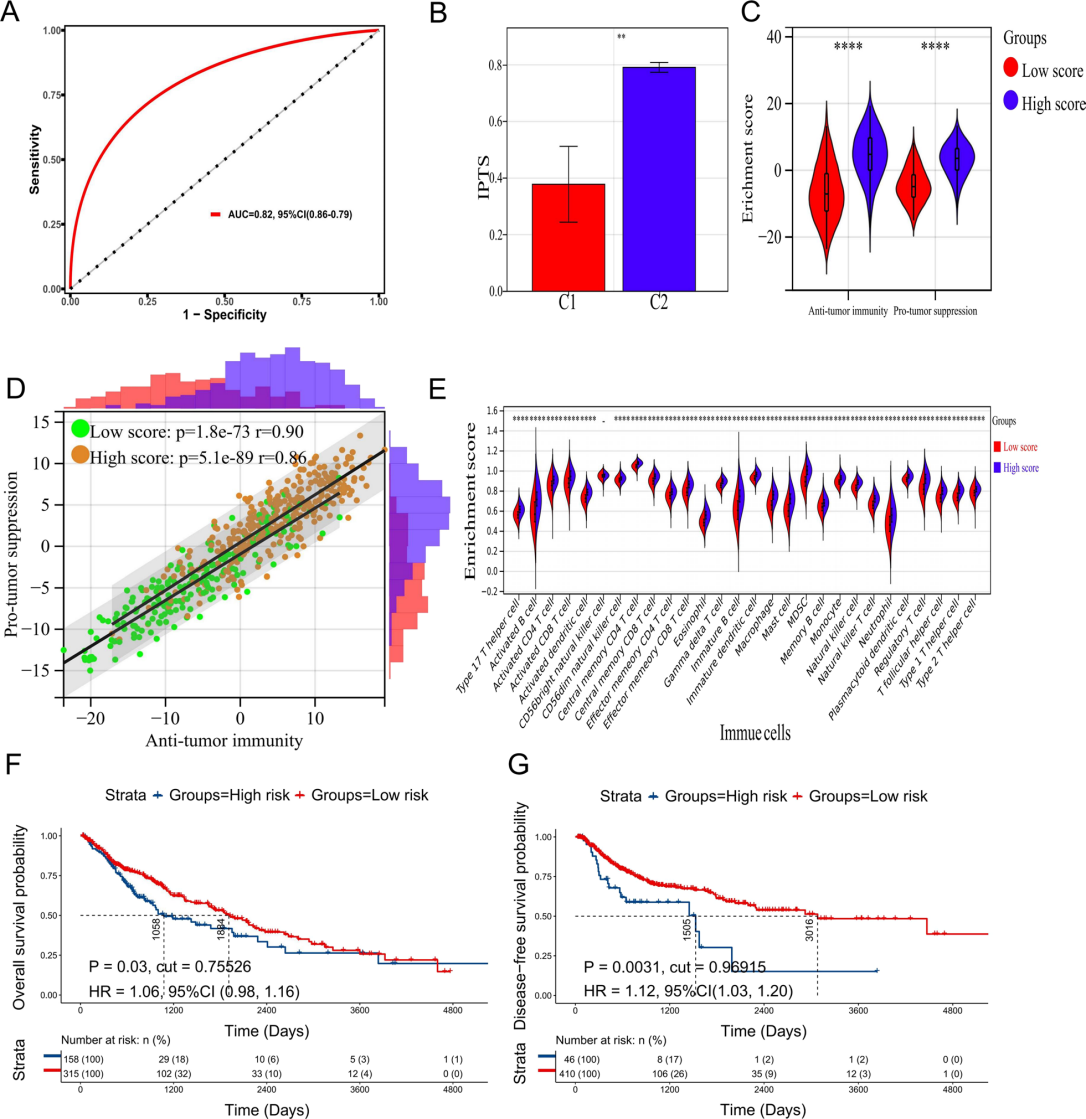
Molecular typing based on IPTS prediction model can replace NMF typing. **A** ROC curves plotted based on the IPTS and NMF typing; **B** histogram of IPTS between C1 and C2 subtypes. The IPTS of type C2 was significantly higher than that of type C1; **C** violin plot between enrichment scores of anti- and pro-tumor immunity in both high and low IPTS groups; **D** scatter plot of correlation between enrichment scores of anti- and pro-tumor immunity in both high and low IPTS groups; **E** violin plot between enrichment scores of 28 types of immune cells in both high and low IPTS groups; **F** survival analysis showed the low score group had better OS than the high score groups (for IPTS = 0.75526); **G** survival analysis showing better DFS in low compared to high score groups (for IPTS = 0.96915); **P < 0.01, ****P < 0.0001

Through analysis and drawing the ROC curve, the AUC was found to be 0.82 (95% CI = 0.86–0.79) (Fig. 4A), and the cutoff value corresponding to the maximum Youden index showed a prediction probability of 50% in the nomogram, i.e., 0.6369. At this point, the prediction sensitivity of the ROC curve was 0.8038, and the specificity was 0.7189, indicating that the IPTS model could well predict the NMF typing of patients with LUSC. Moreover, the IPTS of patients in type C2 was significantly higher than that of patients in type C1 (*P* = 0.0026, Fig. 4B), and the number of patients in type C2 with higher IPTS was significantly higher than that of patients in type C1 (*P* < 0.0001, Table 1). It can be preliminarily concluded that molecular typing based on the IPTS could well predict NMF typing.

To further clarify whether IPTS subtyping could replace NMF typing, we also performed difference analysis and correlation analysis. As shown in Fig. 4C, the enrichment scores of anti-tumor immunity (*P* < 0.0001) and pro-tumor immunity (*P* < 0.0001) in patients with high IPTS were significantly higher than those in patients with low IPTS. Furthermore, there were significant correlations between the enrichment scores of anti- and pro-tumor immunity both in the high and low IPTS groups (Fig. 4D). The correlation coefficient of the high score group was 0.86 (*P* = 5.1e−89), while the correlation coefficient of the low score group was 0.90 (*P* = 1.8e−73). In addition, except for CD56 bright NK cells (*P* = 0.07), the enrichment scores of TIICs in the high IPTS group were significantly higher than those in the low IPTS groups (*P* < 0.0001, Fig. 4E). Therefore, molecular typing based on IPTS can replace NMF typing and has potential therapeutic value for clinical application.

Since our prediction model was constructed based on genes that affect the OS and DFS of patients, we performed survival analysis to evaluate the prognostic predictive value of this model. Furthermore, we assessed whether the model could compensate for the missing prognostic prediction function of the NMF typing. The results suggested that when the optimal cutoff value was used instead of the cutoff value of the ROC curve, the low IPTS group had better OS (HR = 1.06, 95% CI = 0.98–1.16, *P* = 0.03, Fig. 4F) and DFS (HR = 1.12, 95% CI = 1.03–1.20, *P* = 0.0027, Fig. 4G) than the high IPTS group. The prediction model could predict the prognosis of patients under certain conditions. For example, when predicting OS, the best cutoff value of IPTS was 0.75526; while when predicting DFS, the best cutoff value of IPTS was 0.96915 in the training set.

### Immune infiltration prediction model predicts immune efficacy of immunotherapy and the potential therapeutic value of five anti-tumor drugs

**Fig. 5:**
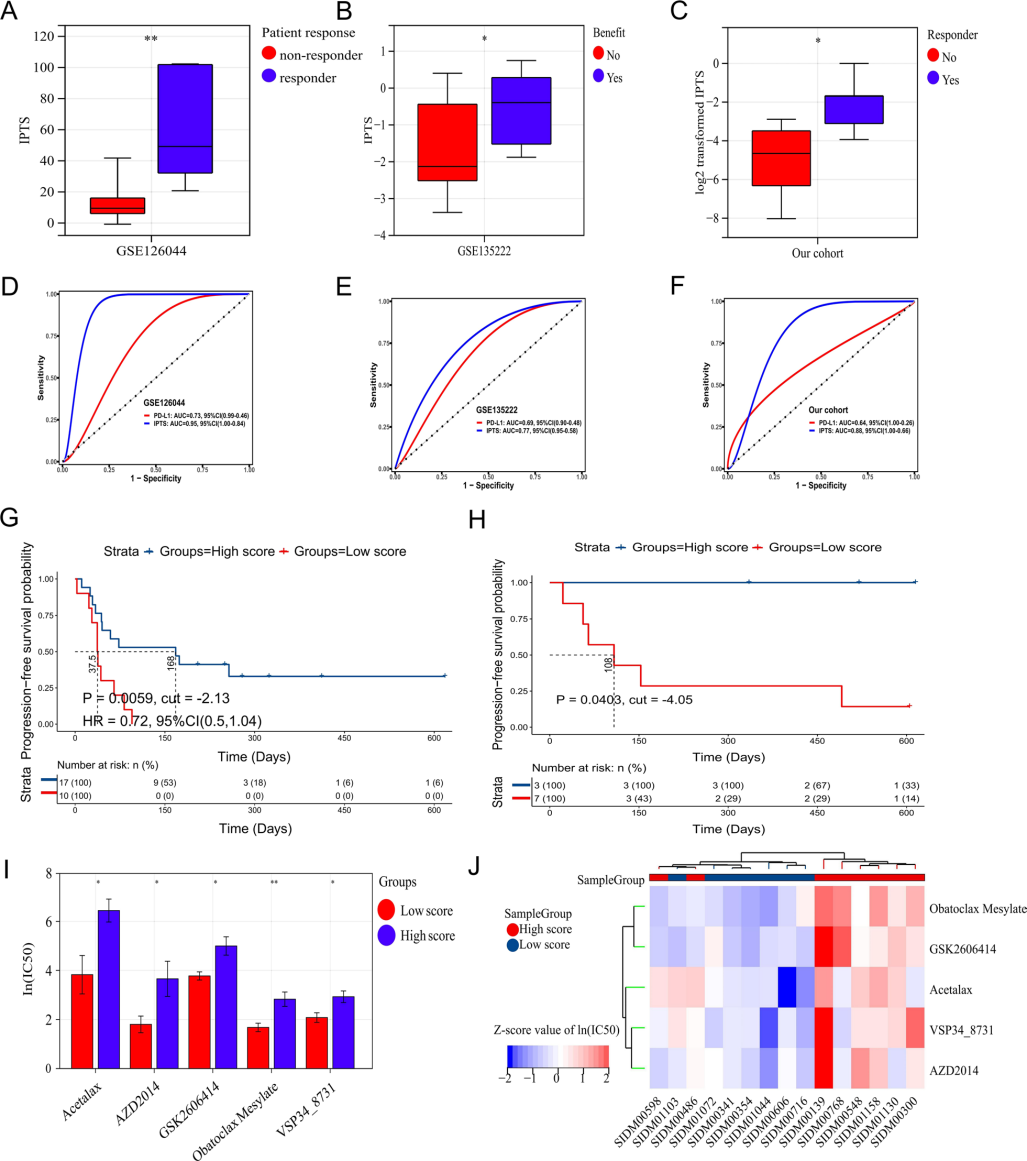
Immune infiltration prediction model predicts immune efficacy of immunotherapy vs five anti-tumor drugs. **A**–**C** Boxplots of IPTS between responder and non-responder groups in the validation set **A** GSE126044, **B** GSE135222, and **C** our independent LUSC cohort. The IPTS was significantly higher in the responder/benefit group than in the non-responder/no-benefit group; **D**–**F** ROC curve plot based on IPTS and immune response of patients in validation dataset **D** GSE126044, **E** GSE135222, and **F** our independent LUSC cohort; **G**, **H** survival curve plot according to IPTS, PFS time, and survival status of patients in the validation dataset **G** GSE135222 and **H** our independent LUSC cohort; **I** histogram based on IPTS molecular typing and the IC_50_ of five anti-tumor drugs; **J** heatmap based on IPTS molecular typing and the IC_50_ of five anti-tumor drugs. The IC_50_ of these drugs in the high score group was generally higher than in the low score group. *P < 0.05, **P < 0.01

The above survival analysis results indicated that patients with high IPTS have worse prognoses. However, to our knowledge, these patients theoretically benefit from immunotherapy due to the high degree of immune cell infiltration. Therefore, we analyzed and verified this view via two data sets in patients that received immunotherapy. In the NSCLC cohort (GSE126044) receiving anti-PD-1 antibody immunotherapy, patients who responded to immunotherapy had significantly higher IPTSes than patients who did not respond to immunotherapy (*P* = 0.0032) (Fig. 5A) with a ROC AUC of 0.95 (95% CI = 1.00–0.84), while the ROC AUC of PD-L1 was 0.73 (95% CI = 0.99–0.46, Fig. 5D) indicating that IPTS has a great predictive effect for the efficacy of immunotherapy in this dataset. In another NSCLC cohort (GSE135222), patients who benefited from immunotherapy had higher IPTSes than those who did not benefit from immunotherapy (*P* = 0.0451) (Fig. 5B) with a ROC AUC of 0.77 (95% CI = 0.96–0.58), and it was also larger than that of PD-L1 of 0.69 (95% CI = 0.90–0.48, Fig. 5E), suggesting that the IPTS has a good predictive value of immunotherapy efficacy in this cohort. In addition, this dataset reported the prognostic information on the PFS of patients. By taking the optimal cutoff value (IPTS = − 2.13), the PFS of patients with high IPTS after immunotherapy was better than that of patients with low IPTS (HR = 0.72, 95% CI = 0.5–1.04, *P* = 0.0059, Fig. 5G), which also showed a good predictive value of immunotherapy efficacy and a certain predictive value of survival prognosis.

In our independent LUSC cohort, there are two patients received complete response (CR), 3 patients received partial response (PR), 4 patients received stable disease (SD), and 1 patient received progression of disease (PD) according to response evaluation criteria in solid tumours (RECIST, v1.1). All ten patients were divided into two groups, responder group (CR + PR) and non-responder group (SD + PD) (Additional file 1: Fig. S3). The results showed that patients in responder group had significantly higher IPTSes than patients in non-responder group (*P* = 0.0325) (Fig. 5C) with a ROC AUC of 0.88 (95% CI = 1.00–0.66), which was larger than that of PD-L1 of 0.64 (95% CI = 1.00–0.26, Fig. 5F). The PFS of patients with high IPTS after immunotherapy was better than that of patients with low IPTS (*P* = 0.0403, Fig. 5H) by taking the best cutoff value (IPTS = − 4.05). Therefore, it could be confirmed that the constructed immune infiltration prediction model has predictive value for immune efficacy, i.e., immunotherapeutic efficacy could be better for patients with high IPTS.

From the above analysis, patients with low IPTS were not likely to benefit from immunotherapy. For this subtype of patients, we initially screened other anti-tumor drugs that may have a curative effect through the GDSC database. The analysis results showed that the IC_50_ of the five anti-tumor drugs acetalax (*P* = 0.0168), AZD2014 (*P* = 0.0416), GSK2606414 (*P* = 0.0145), obatoclax mesylate (*P* = 0.0061), and VSP34_8731 (*P* = 0.0163) were higher in the high IPTS group than in the low IPTS group in LUSC cell lines (Fig. 5I). Moreover, it can be seen from the heat map (Fig. 5J) that the IC_50_ value of the high IPTS group was generally higher than that of the low IPTS group, indicating that patients in the low IPTS group might be more sensitive to these five drugs.

### IPTS positively correlates with immune microenvironment score and expression of immune-related genes signatures

**Fig. 6:**
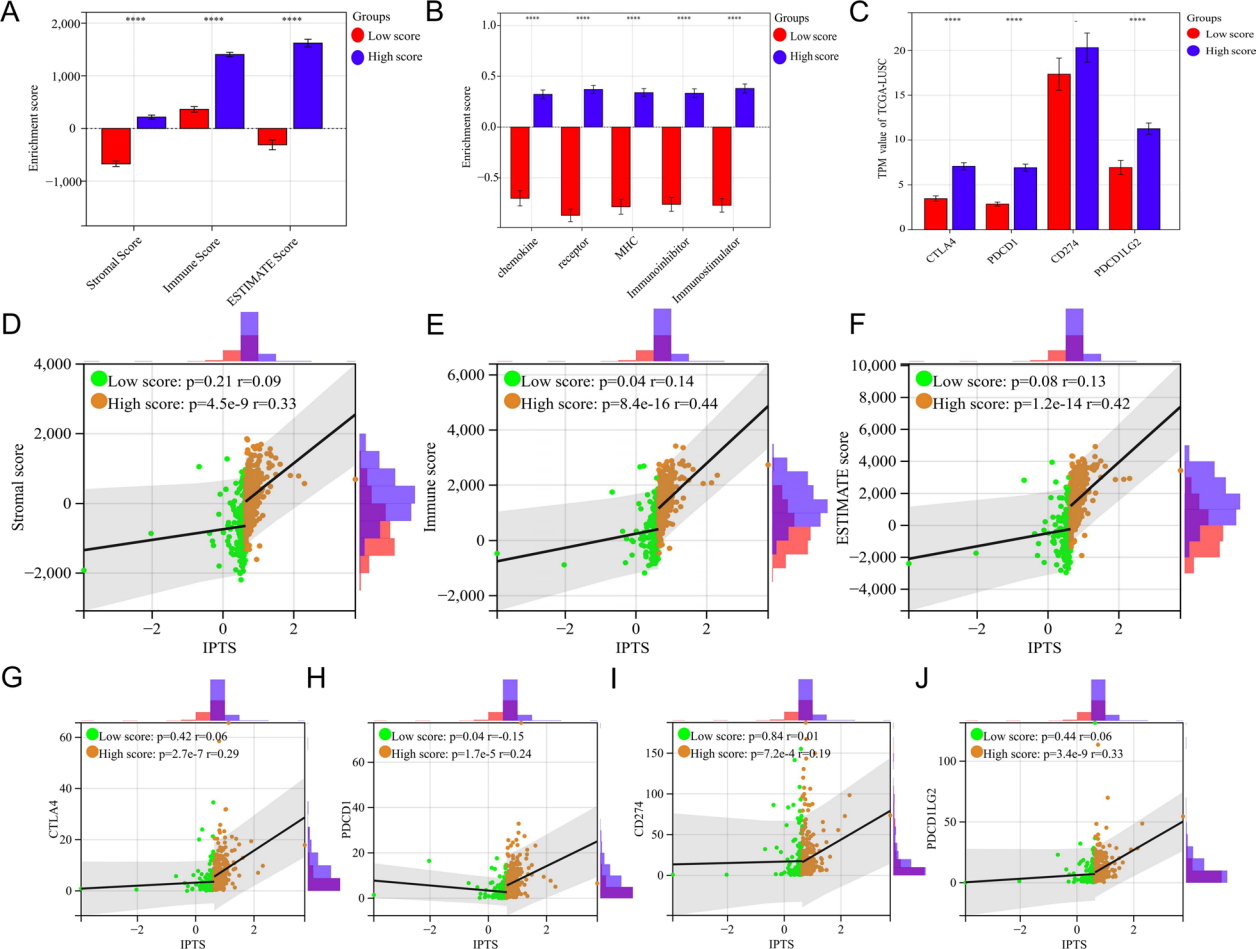
IPTS positively correlates with immune microenvironment score and expression of immune-related genes signatures. **A** Histograms based on IPTS molecular typing and tumor microenvironment (TME)-related enrichment scores; **B** histograms based on IPTS molecular typing and enrichment scores of the five immune molecular typing; **C** histogram based on IPTS molecular typing and TPM values of four immune checkpoints sequenced in TCGA-LUSC database; **D**–**F** Scatter plot of correlations based on IPTS and **D** stromal scores, **E** immune scores, and **F** ESTIMATE scores. **G**–**J** Scatter plot of correlations based on IPTS and expression of **G** CTLA-4, **H** PD-1, **I** PD-L1, and **J** PD-L2. ****P < 0.0001

The immune microenvironment scores of 501 patients with LUSC in the training set were evaluated using grouping analysis of IPTS molecular typing. The results showed that the stromal score (*P* < 0.0001), immune score (*P* < 0.0001), and ESTIMATE score (*P* < 0.0001) in the high IPTS group were significantly higher than those in the low IPTS group (Fig. 6A), suggesting that higher stromal cell levels and infiltration levels of immune cells in the high compared to low IPTS groups. This further confirms that tumors in the high IPTS subtype tended to be “hot tumors”. Moreover, through correlation analysis, IPTS was significantly positively correlated with the stromal score (*P* = 2.0e−22, *r* = 0.42, Additional file 1: Fig. S4A), immune score (*P* = 2.2e−31, *r* = 0.49, Additional file 1: Fig. S4B), and ESTIMATE score (*P* = 1.9e−30, *r* = 0.48, Additional file 1: Fig. S4C). Meanwhile, the correlation analysis of IPTS molecular typing showed that only the immune score (low score: *P* = 0.04, *r* = 0.14; High score: *P* = 8.4e−16, *r* = 0.44, Fig. 6E) was statistically significant in the low IPTS group, whereas there was no significant difference in stromal score (Low score: *P* = 0.21, *r* = 0.09; High score: *P* = 4.5e−9, *r* = 0.33, Fig. 6D) and ESTIMATE score (low score: *P* = 0.08, *r* = 0.13; High score: *P* = 1.2e−14, *r* = 0.42, Fig. 6F), suggesting that the three immune microenvironment scores were mainly positively correlated with IPTS in the high score group.

In addition, we analyzed the five immune molecular typing scores of the samples in the training set by ssGSEA. The detailed gene signatures of the immune molecular typing gene markers are listed in Additional file 5: Table S4. Similarly, according to the IPTS molecular typing analysis, the results showed that the enrichment scores of chemokines (*P* < 0.0001), receptors (*P* < 0.0001), MHC molecules (*P* < 0.0001), immuno-inhibitors (*P* < 0.0001), and immuno-stimulators (*P* < 0.0001) in the high IPTS group were significantly higher than those in the low IPTS group (Fig. 6B). Furthermore, the four targets of immunotherapy drugs commonly used in clinical practice all relate to immuno-inhibitors. Therefore, the differences in expression values of the four immuno-inhibitors CTLA-4, PD-1 (PDCD1), and its two ligands PD-L1 (CD274) and PDL-2 (PDCD1LG2) were analyzed between the IPTS groups in the training set. The results showed that the expressions of *CTLA-4* (*P* < 0.0001), *PD-1* (*P* < 0.0001), and *PD-L2* (*P* < 0.0001) in the high IPTS group were significantly higher than those in the low IPTS group, whereas that of *PD-L1* expression was insignificant (*P* = 0.22) (Fig. 6C). Further correlation analysis showed that IPTS was significantly positively correlated with the expressions of *CTLA4* (*P* = 2.6e−11, *r* = 0.29, Additional file 1: Fig. S4D), *PD-1* (*P* = 1.8e−8, *r* = 0.25, Additional file 1: Fig. S4E), *PDL1* (*P* = 8.3e−3, *r* = 0.12, Additional file 1: Fig. S4F), and *PD-L2* (*P* = 6.4e−9, *r* = 0.26, Additional file 1: Fig. S4G). The results of IPTS molecular typing correlation analysis showed a significant negative correlation of *PD-1* expression with IPTS in the low score group (*P* = 0.04, *r* = − 0.15, Fig. 6H), whereas those of the other three immuno-inhibitors were not statistically significant. In the high score group, the expressions of the four immuno-inhibitors *CTLA-4* (*P* = 2.7e−7, *r* = 0.29, Fig. 6G), *PD-1* (*P* = 1.7e−5, *r* = 0.24, Fig. 6H), *PD-L1* (*P* = 7.2e−4, *r* = 0.19, Fig. 6I), and *PD-L2* (*P* = 3.4e−9, *r* = 0.33, Fig. 6J) were positively correlated with IPTS. To a certain extent, the above results further provide a theoretical basis for better immunotherapy efficacy in patients with high IPTS.

### High IPTS involved in immune-related signaling pathways

**Fig. 7:**
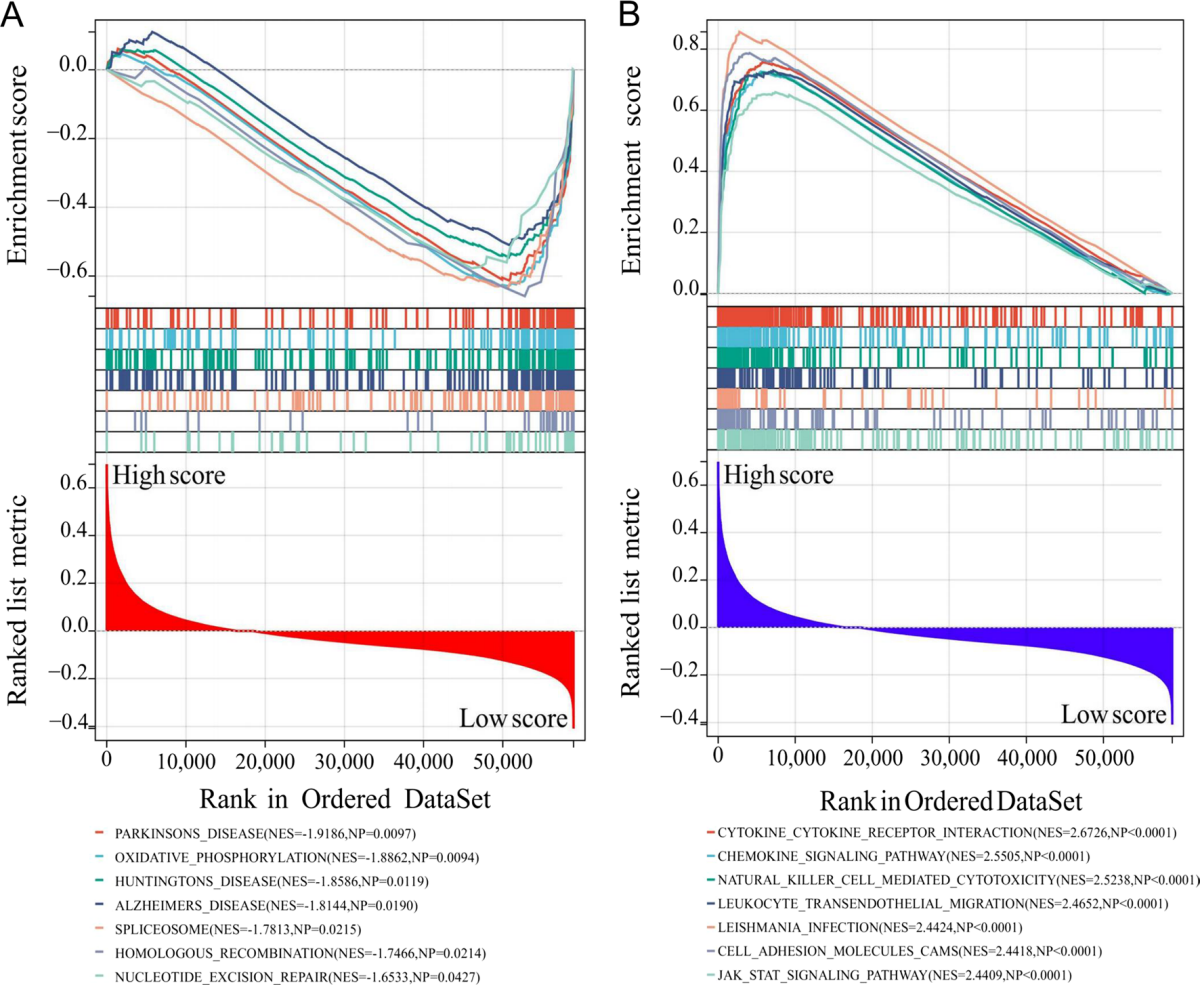
High IPTS involved in immune-related signaling pathways. **A**, **B** The top seven pathways with biological significance in GSEA in the **A** low IPTS and **B** high IPTS groups ranked by NES

The results of GSEA indicated that the IPTS was mainly related to Parkinson’s disease (NES = − 1.9186, NP = 0.0097), oxidative phosphorylation (NES = − 1.8862, NP = 0.0094), Huntington’s disease (NES = − 1.8586, NP = 0.0119), Alzheimer’s disease (NES = − 1.8144, NP = 0.0190), spliceosome (NES = − 1.7813, NP = 0.0215), homologous recombination (NES = − 1.7466, NP = 0.0214), nucleotide excision repair (NES = − 1.6533, NP = 0.0427), etc. (Fig. 7A). In contrast, high IPTS was mainly associated with cytokine-cytokine receptor interaction (NES = 2.6726, NP < 0.0001), chemokine signaling pathway (NES = 2.5505, NP < 0.0001), natural killer cell mediated cytotoxicity (NES = 2.5238, NP < 0.0001), leukocyte trans-endothelial migration (NES = 2.4652, NP < 0.0001), Leishmania infection (NES = 2.4424, NP < 0.0001), cell adhesion molecules (NES = 2.4418, NP < 0.0001), JAK-STAT signaling pathway (NES = 2.4409, NP < 0.0001), etc. (Fig. 7B). These results indicate that low IPTS was mainly involved in disease, genetic, and metabolic-related signaling pathways, while high IPTS was mainly involved in immune-related signal pathways. All the results of GSEA are detailed in Additional file 6: Table S5.

## Discussion

The past decade witnessed great strides in cancer diagnosis and treatment. However, progress in improving the survival of patients with lung cancer has been slow, with an average 5-year survival rate of only 10–20% in most countries [44, 45]. In recent years, immunotherapy has achieved promising results in clinical practice. The latest research suggested a 5-year survival rate as high as 23.2% in patients with advanced NSCLC using anti-PD-1 antibodies as the first-line treatment [46]. Furthermore, the 5-year survival rate of patients treated with anti-PD-1 antibodies as a second-line treatment has also reached 16% [47], which is twice as high as that of traditional treatments. Nevertheless, several studies have revealed that only ~ 20% of patients with NSCLC could benefit from ICI therapy [48, 49], which illustrates the importance of selecting patients that will potentially benefit. Recently, Tian et al. [50] conducted an immune subgroup analysis study on NSCLC including LUSC, lung adenocarcinoma, and lung adenosquamous carcinoma, and found that mast cell types had a significant impact on the prognosis of patients with LUAD while the presence of monocytes was significantly associated with OS in patients with LUSC. Furthermore, the authors pointed out that LUSC and LUAD may require independent analysis. This is in accordance with a study reported by Jiang et al. [115] on the prediction of immunotherapy efficacy in NSCLC that also suggested the underlying immune response mechanism between LUAD and LUSC may be different. Therefore, we constructed a prediction model of immunotherapy efficacy to improve the accuracy of screening patients with LUSC for potential benefit from ICI treatment.

Detecting the expression level of PD-L1 is the most commonly used method to predict the efficacy of immunotherapy [51]. Some scholars have previously constructed some efficacy prediction models for tumor immunotherapy, such as the Tumor Immune Dysfunction and Exclusion (TIDE) [52] and the Tumor Inflammation Signature (TIS) [53, 54]. By comparing with PD-L1 expression level to predict the efficacy of immunotherapy, in our independent LUSC cohort and two validation sets, the ROC AUC of IPTS molecular typing was increased by 24%, 22% and 8% respectively compared with that of PD-L1 expression level. The results suggest that the prediction effect of our model is similar to that of TIDE or TIS. However, compared with TIDE, which needs to use whole gene transcriptome data to conduct online prediction, or TIS, which only knows the gene type and does not disclose the relevant calculation equations, and requires a special analysis system, building a IPTS model equation to predict the efficacy of immunotherapy have the advantages of lower cost and more convenience.

In our study, a total of 17 genes were screened to construct a predictive model for immunotherapy efficacy in patients with LUSC, of which 9 genes (*AKAP2*, *GCGR*, *LRRC38*, *MARCO*, *NANOG*, *NTSR1*, *PF4*, *RP1*, and *TMEM236*) were gene signatures of C1, and 8 genes (*ALOX5*, *FCGR2A*, *KCNQ3*, *NLRP12*, *SCARF1*, *SIGLEC12*, *TGM2*, and *VSTM1*) were gene signatures of C2. In previous studies, some of these genes have been associated with cancer progression and prognosis. Among these, *AKAP2* was found to be upregulated in ovarian cancer, and promotes cancer cell growth as well as migration [55]. Increased expression of *AKAP2* has been linked to metastatic prostate cancer, while knocking down its expression could significantly reduce the tumorigenicity and metastatic ability of prostate cancer cells [56]. *GCGR* was found to be an independent prognostic factor for OS in patients with NSCLC [57]. The protein encoded by *MARCO* is a member of the scavenger receptor family. It has been shown that targeting the scavenger receptor MARCO with antibodies reduces tumor growth and metastasis in murine tumor models of melanoma, colon cancer, and breast cancer [58]. Furthermore, the homeobox-domain transcription factor NANOG, a key regulator of embryonic development and cellular reprogramming, is ubiquitously expressed in human cancers [59]. Its overexpression has been linked to a worse prognosis in lung cancer [60]. *NTSR1* is reportedly expressed in 40% of lung tumors, and its expression is a negative prognostic marker in patients with surgically resected stage I lung adenocarcinoma [61]. PF4 is a cancer-enhancing endocrine signal, and its overexpression in tumors is associated with reduced OS in patients with lung cancer [62]. As six of the nine genes associated with low immune cell infiltration (type C1) were involved in the pathogenesis, malignant transformation, and progression of a variety of cancers, including LUSC, as well as showing significant correlations with patient survival and prognosis, the findings of our bioinformatics analysis are meaningful to an extent.

Among the 8-gene signature of high immune cell infiltration (type C2), *ALOX5* has been found to promote gastric cancer growth and attenuate chemotherapy toxicity [63], while in breast cancer, ALOX5 activation is associated with *HER2* expression as well as mediates breast cancer growth and migration [64]. Recent studies have reported that the polymorphism of *FCGR2A* expression is associated with an increased risk of lung cancer [65]. NLRP12 is a key factor in maintaining intestinal homeostasis and preventing colorectal tumors [66]. Higher SCARF1 expression in hepatocellular carcinoma tumor tissues was highly prognostic of better OS, DFS and PFS [67]. High frequency of *SIGLEC12* expression in advanced colorectal cancer cohort and correlation with OS [68]. TGM2 has been shown to enhance the migration and invasion of lung cancer cells [69]. *TMEM236* has the potential to be a potential novel diagnostic biomarker for colorectal cancer [70]. Downregulated in bone marrow cells from leukemia patients, VSTM1 may become a diagnostic and treatment target [71]. Only the three remaining genes, *KCNQ3*, *LRRC38* and *RP1*, were rarely reported in any cancer research, and thus show potential value for research in LUSC.

Among these 17 genes, 13 genes were reported to be associated with immune-related pathways. The pathway with the largest number of associated genes is the mitogen-activated protein kinases (MAPKs) signaling pathway. Eight genes could regulate it, and they are *AKAP2* [72], *ALOX5* [63], *GCGR* [73], *NLRP12* [74], *NTSR1* [75], *PF4* [76], *SIGLEC12* [68] and *TGM2* [77]*.* Wnt/β-catenin signaling pathway could be regulated by *AKAP2* [55], *ALOX5* [78] and *TGM2* [79]*.* PI3K/AKT/mammalian target of rapamycin (mTOR) signaling pathway could be regulated by *ALOX5* [80], *SCARF1* [81] and *TGM2* [82]*.* There are seven genes involved in the regulation of nuclear transcription factor-κB (NF-κB) signaling pathway, such as *ALOX5* [83], *MARCO* [84], *NANOG* [85], *NLRP12* [74], *NTSR1* [75], *TGM2* [86], and VSTM1 [87]. Toll-like receptors (TLRs) signaling pathway could be regulated by *FCGR2A* [88], *MARCO* [89], *NANOG* [90], *NLRP12* [74], and *PF4* [91]*.* Six genes could involved in the regulation of janus kinase/signal transducer and activator of transcription (JAK/STAT) signaling pathway, such as *ALOX5* [78, 92], *MARCO* [93], *NLRP12* [94], *PF4* [95], *SCARF1* [81], and *TGM2* [96]*.* In addition, some genes have other immune-related functions. For instance, ALOX5 contributes to the recruitment and activation of macrophages thereby adding to the role of macrophages in a dynamically changing tumor environment [97]. *FCGR2A* encodes the receptor protein on the surface of immune cells, which can transmit activation signals to cells through its tyrosine-based activation motif [98]. Antibodies targeting MARCO in NSCLC restore the anti-tumor activity of T cells and NK cells by polarizing suppressor macrophages [99]. NLRP12 plays critical roles in balancing T cell response to control overt activation and maintain cellular homeostasis [100]. SCARF1 mediates the clearance of apoptotic cells and prevents autoimmunity [101]. *SIGLEC12* encodes one of the CD33-related SIGLEC family of signaling molecules in immune cells [102]. The binding of the TGM2 mediated crosslinked fibrinogens to un-stimulated endothelial cells can assemble leukocytes, platelets or fibrin, and promote inflammation [103]. Only the four remaining genes, *KCNQ3*, *LRRC38*, *RP1* and *TMEM236* were rarely reported in any immue-related research, which provides new ideas for follow-up studies based on these four genes, especially in the immunological research related to LUSC.

ICIs enhance T cell activity by blocking CTLA-4, PD-1, or PD-L1 to achieve an anti-tumor effect. The high expression of *CTLA-4*, *PD-1*, and *PD-L1/PD-L2* has been positively correlated with the efficacy of immunotherapy, which has a certain value for therapeutic prediction [104]. By exploring the relationship between IPTS and the expression of *CTLA-4*, *PD-1*, *PD-L1*, and *PD-L2*, we found that the expression of four immuno-inhibitors was significantly positively correlated with the IPTS in the high score group. In addition, the difference analysis of immune molecular typing between the two IPTS subgroups (either high or low scores) revealed that the enrichment scores of chemokines, chemokine receptors, MHC molecules, immuno-inhibitors, and immuno-stimulators in patients with high IPTS were significantly higher than those in patients with low IPTS. These findings further indicated evident differences in the immune microenvironment between these two subtypes, with tumors in the high score group more likely to be “hot tumors”.

Our study found that patients with high IPTS had a worse prognosis than those with low IPTS in the training set (patients not receiving immunotherapy), while in the validation set GSE135222 and our LUSC cohort (patients receiving immunotherapy), this situation had been reversed. In other words, patients with high IPTS were more likely to benefit from immunotherapy than those with low IPTS. As for patients with low IPTS, we further explored the correlation between IPTS and anti-tumor drug efficacy, and found that the IC_50_ of five drugs (i.e., acetalax, AZD2014, GSK2606414, obatoclax mesylate, and VSP34_8731) in LUSC cells with high IPTS was higher than that in cells with low IPTS, suggesting that patients with low IPTS might be sensitive to these drugs. Among them, acetalax, also known as oxyphenisatin acetate, has shown antitumor activity in mouse xenograft models by inducing tumor necrosis factor (TNF) α expression and TNFR1 degradation, indicating autocrine TNF α-mediated apoptosis. AZD2014 is a mTOR inhibitor [105]. mTOR is a key kinase of PI3K/AKT/mTOR signaling pathway, which can regulate the tumor cell proliferation, differentiation, apoptosis and other processes. Previous studies have shown that mTOR signaling pathway has a significant regulatory effect on immune function and T cell differentiation by integrating various microenvironment signals [106, 107]. AZD2014 has been proved to have dramatic anti-tumor effects in phase II clinical trials for breast cancer [108] and hepatocellular carcinoma [109]. As a protein kinase R-like endoplasmic reticulum kinase (PERK) inhibitor, GSK2606414 can significantly inhibit the PERK dependent signaling pathway in human colorectal adenocarcinoma cell line HT-29 and human neuroblastoma cell lines SH-SY5Y, which can promote apoptosis by inducing endoplasmic reticulum stress [110, 111]. The pan-Bcl-2 inhibitor Obatoclax can sensitize hepatocellular carcinoma cells to promote the anti-tumor efficacy in combination with ICIs, for Obatoclax can sensitize T cell mediated killing by promoting T cell activation and the expression of effector cytokines in spleen and tumor [112]. VSP34, as a type III phosphatidylinositol kinase, is a key protein in the process of autophagy [113]. Recently, Noman et al. [114] reported that VSP34 regulated the TME through its kinase activity, and VSP34 protein knockdown or VSP34 kinase activity inhibition could transform tumors from “cold tumors” to “hot tumors” to enhance the effect of ICIs. As an inhibitor targeting VSP34, VSP34_8731 has the potential to realize the transition from C1 tumors to C2 tumors by increasing the infiltration of immune cells into tumor tissues. It can be concluded from the above studies that these five drugs have the effects of regulating immune process thereby promoting tumor cell apoptosis, and it might be the reason that the LUSC cell lines with low IPTSes may be more sensitive to these five antitumor drugs. This also demonstrates the feasibility of our study in using high and low immune cell infiltration typing for patients with LUSC as a measure of immunotherapy efficacy, and our findings provided a theoretical basis for the selection of treatment methods in patients with LUSC, and also put forth a new treatment scheme with potential curative effect for patients with poor outcomes after immunotherapy.

Our study presented a potential new method for predicting the efficacy of immunotherapy in LUSC. Nevertheless, there are still some limitations that should not be ignored. First, based on the data from public databases, the internal mechanism still needs experimental verification. Through functional enrichment analysis, it was found that the high IPTS groups involved the regulation of multiple pathways related to tumor occurrence and development, which requires follow-up molecular mechanism research. Second, due to the different sequencing platforms of the training set (TCGA-LUSC) and validation sets (GSE126044 and GSE135222) giving rise to different sequencing backgrounds and normalization methods, it is difficult to obtain the best IPTS value suitable for all data sets to distinguish high or low immune cells infiltration. Therefore, the initial IPTS threshold should be obtained through small sample testing, and then corrected by conducting a large-scale prospective clinical study. Furthermore, whether a high IPTS could become a predictor of immunotherapy efficacy also needs to be further confirmed by large-scale prospective clinical trials. Third, regarding anti-tumor drug treatment, the number of LUSC cell lines in the GDSC database is relatively small at only 15. To maximize the test efficiency, we grouped them as high and low IPTS groups according to 1:1; hence, there is likely to be a certain bias. The results of this study may still provide theoretical support for the treatment of LUSC with anti-tumor drugs.

## Conclusion

In conclusion, we constructed a model containing 17 genes to predict the efficacy of immunotherapy for patients with LUSC based on bioinformatics analysis on the TCGA database. The prediction effect of the model was verified in two independent cohorts in the GEO database. The IPTS molecular typing positively correlated with both the degree of tumor immune cell infiltration and the efficacy of immunotherapy with potential prognostic value. This study provides a new method for predicting the efficacy of immunotherapy for LUSC, which may have potential clinical prospects.

## Supplementary Information

**Additional file 1: Figure S1.** The NMF rank survey at a rank of 2 to 10. **Figure S2.** All heatmaps of the training set with the number of clusters ranged from 2 to 10. **Figure S3.** The ten patients’ best response evaluation in our independent LUSC cohort according to response evaluation criteria in solid tumours (RECIST, v1.1). **Figure S4.** Correlation analysis between IPTS and (A) stromal score, (B) immune score, (C) ESTIMATE score, (D) CTLA4 TPM value, (E) PDCD1 (PD-1) TPM value, (F) CD274 (PD-L1) TPM value, and (G) PDCD1LG2 (PD-L2) TPM value.

**Additional file 2: Table S1.** The primers and their seqences of  17 genes and GAPDH.

**Additional file 3: Table S2.** The main clinical information of TCGA-LUSC, GSE126044, GSE135222 and our independent LUSC cohort.

**Additional file 4: Table S3.** All results of GO/KEGG enrichment analysis with FDR (q value) < 0.05.

**Additional file 5: Table S4.** Five types of immunophenotypes and their corresponding gene signatures.

**Additional file 6: Table S5.** All results of GSEA report for low/high score.

## Data Availability

The datasets in the current study are open to the public at the TCGA (https://portal.gdc.cancer.gov) and GEO (https://www.ncbi.nlm.nih.gov/geo) databases. The original contributions presented in the study are included in the article/Additional files. Further inquiries can be directed to the corresponding author.

## Abbreviations

AUC: Area under the curve
CTLA-4: Cytotoxic T lymphocyte-associated antigen-4
CR: Complete response
DEGs: Differentially expressed genes
DFS: Disease-free survival
FDR: False discovery rate
GDSC: Genomics of drug sensitivity in cancer
GEO: Gene expression omnibus
GO: Gene ontology
GSEA: Gene set enrichment analysis
IC50: 50% inhibitory concentration
ICIs: Immune checkpoint inhibitors
IPTS: Immunophenotyping score
irAEs: Immune-related adverse events
JAK/STAT: Janus kinase/signal transducer and activator of transcription
KEGG: Kyoto encyclopedia of genes and genomes
LASSO: Least absolute shrinkage and selection operator
LUSC: Lung squamous cell carcinoma
MAE: Mean absolute error
MAPK: Mitogen-activated protein kinase
MHC: Major histocompatibility complex
mTOR: Mammalian target of rapamycin
NES: Normalized enrichment score
NF-κB: Nuclear transcription factor-κB
NK: Natural killer
NMF: Non-negative matrix factorization
NP: Normalized P-value
NSCLC: Non-small cell lung cancer
OS: Overall survival
PCA: Principal component analysis
PD: Progression of disease
PD-1: Programmed cell death protein-1
PD-L1: Programmed cell death ligand-1
PERK: Protein kinase R-like endoplasmic reticulum kinase
PFS: Progression-free survival
PR: Partial response
RECIST: Response evaluation criteria in solid tumours
ROC: Receiver operating characteristic
SD: Stable disease
ssGSEA: Single-sample gene set enrichment analysis
TCGA: The Cancer Genome Atlas
TIDE: Tumor Immune Dysfunction and Exclusion
TIICs: Tumor-infiltrating immune cells
TIS: Tumor Inflammation Signature
TLR: Toll-like receptor
TMB: Tumor mutational burden
TNF: Tumor necrosis factor
TPM: Transcripts per kilobase million
tSNE: T-distributed stochastic neighbor embedding
