# Supplementary material for: Construction of a predictive model for immunotherapy efficacy in lung squamous cell carcinoma based on the degree of tumor-infiltrating immune cells and molecular typing
Source: J Transl Med. 2022 Aug 12;20:364. doi: 10.1186/s12967-022-03565-7 (PMC9373274; doi:10.1186/s12967-022-03565-7)
Supplement: Supplementary file 1 — Additional file 1: Figure S1. The NMF rank survey at a rank of 2 to 10. Figure S2. All heatmaps of the training set with the number of clusters ranged from 2 to 10. Figure S3. The ten patients’ best response evaluation in our independent LUSC cohort according to response evaluation criteria in solid tumours (RECIST, v1.1). Figure S4. Correlation analysis between IPTS and (A) stromal score, (B) immune score, (C) ESTIMATE score, (D) CTLA4 TPM value, (E) PDCD1 (PD-1) TPM value, (F) CD274 (PD-L1) TPM value, and (G) PDCD1LG2 (PD-L2) TPM value. [file 12967_2022_3565_MOESM1_ESM.pdf]

Supplemental figures

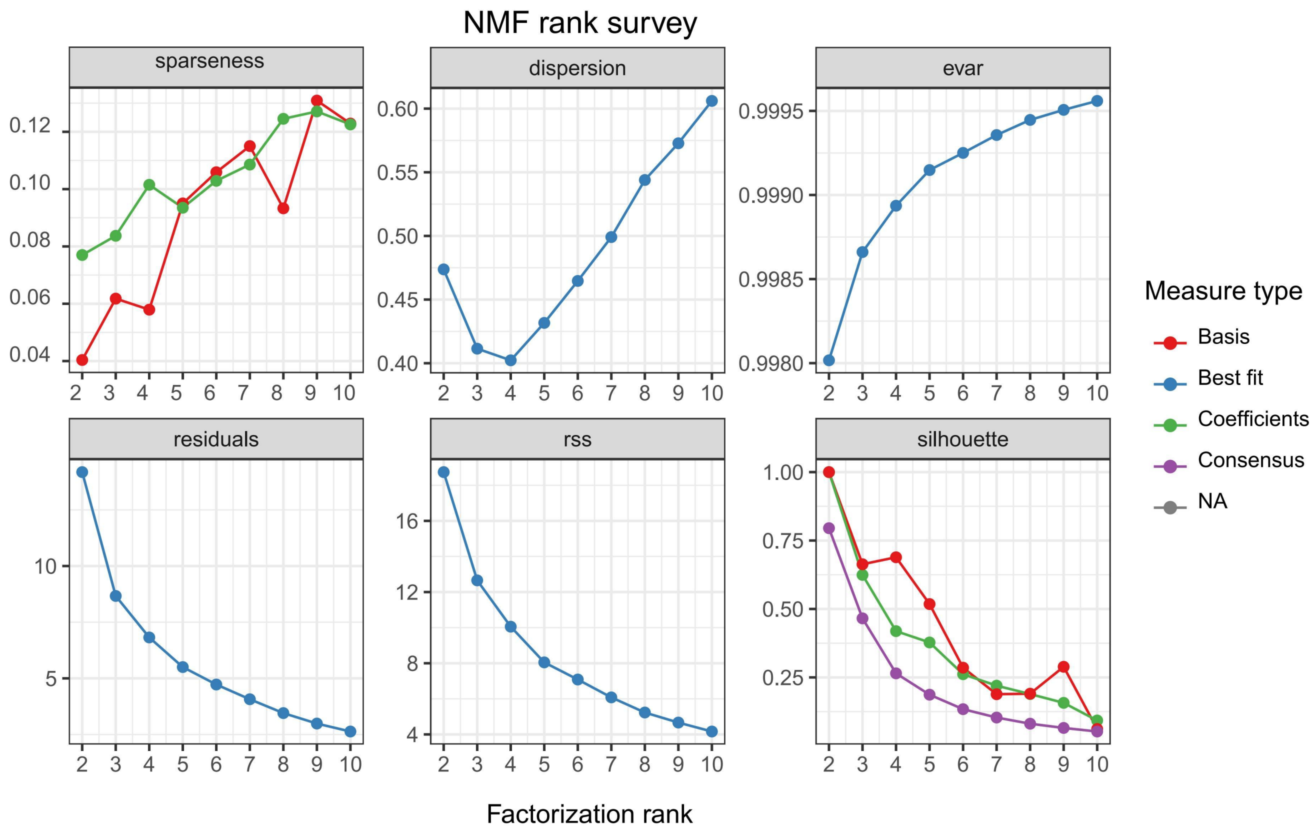

Supplementary Figure 1. The NMF rank survey at a rank of 2 to 10.

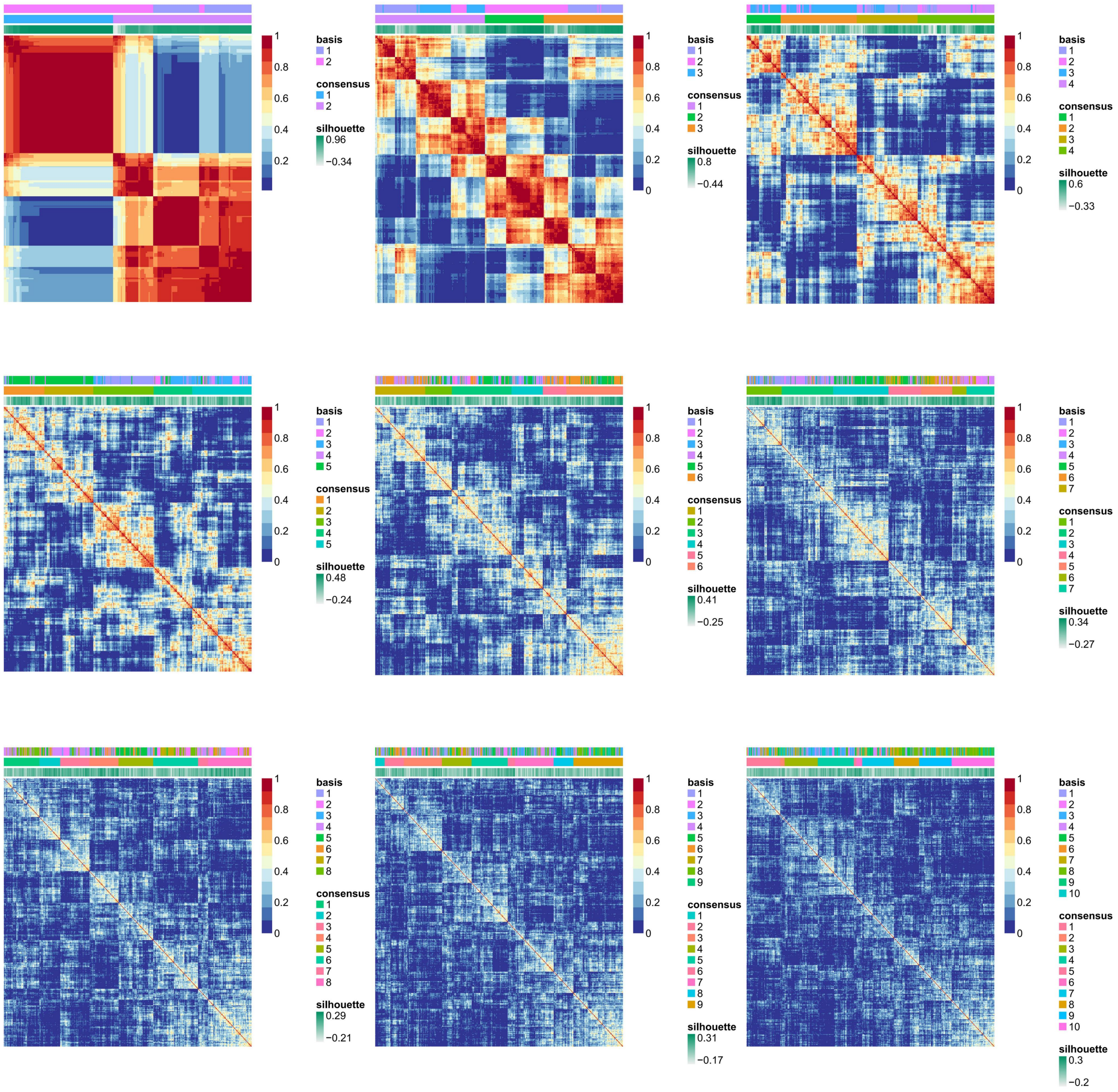

Supplementary Figure 2. All heatmaps of the training set with the number of clusters ranged from 2 to 10.

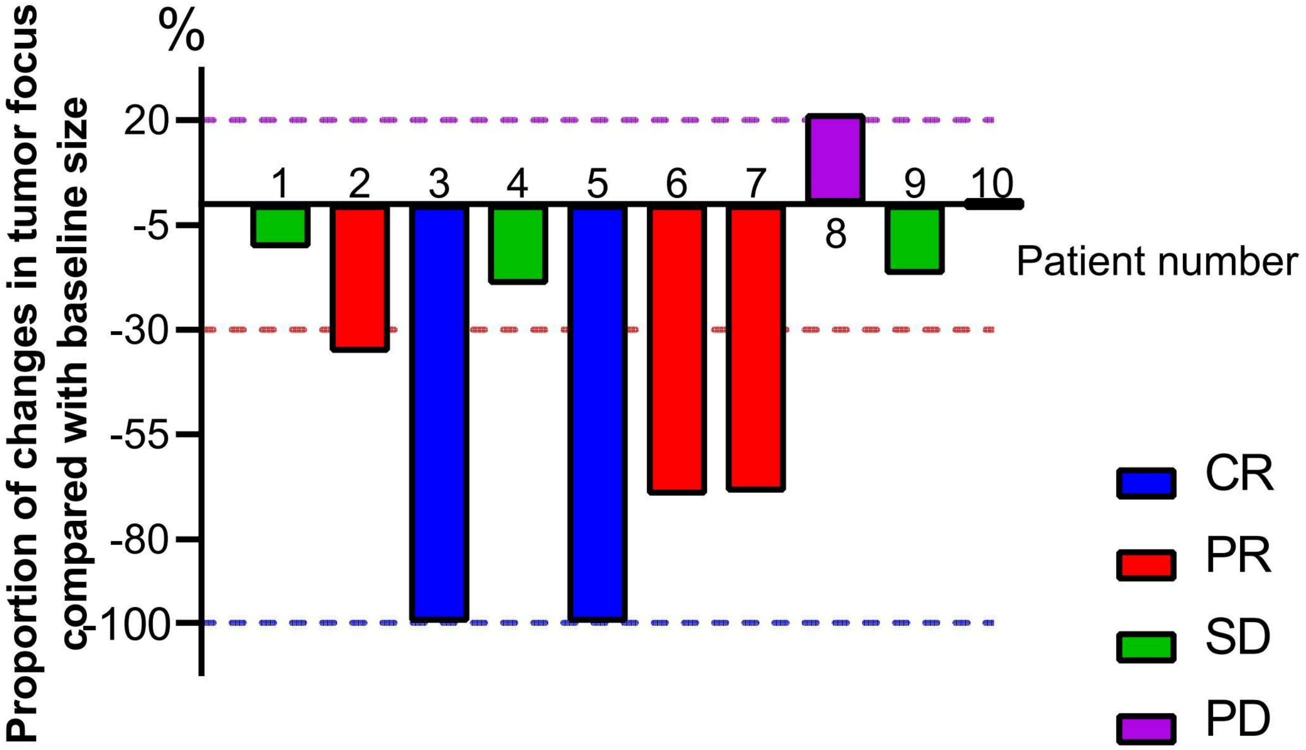

Supplementary Figure 3. The ten patients' best response evaluation in our independent LUSC cohort according to response evaluation criteria in solid tumours (RECIST, v1.1).

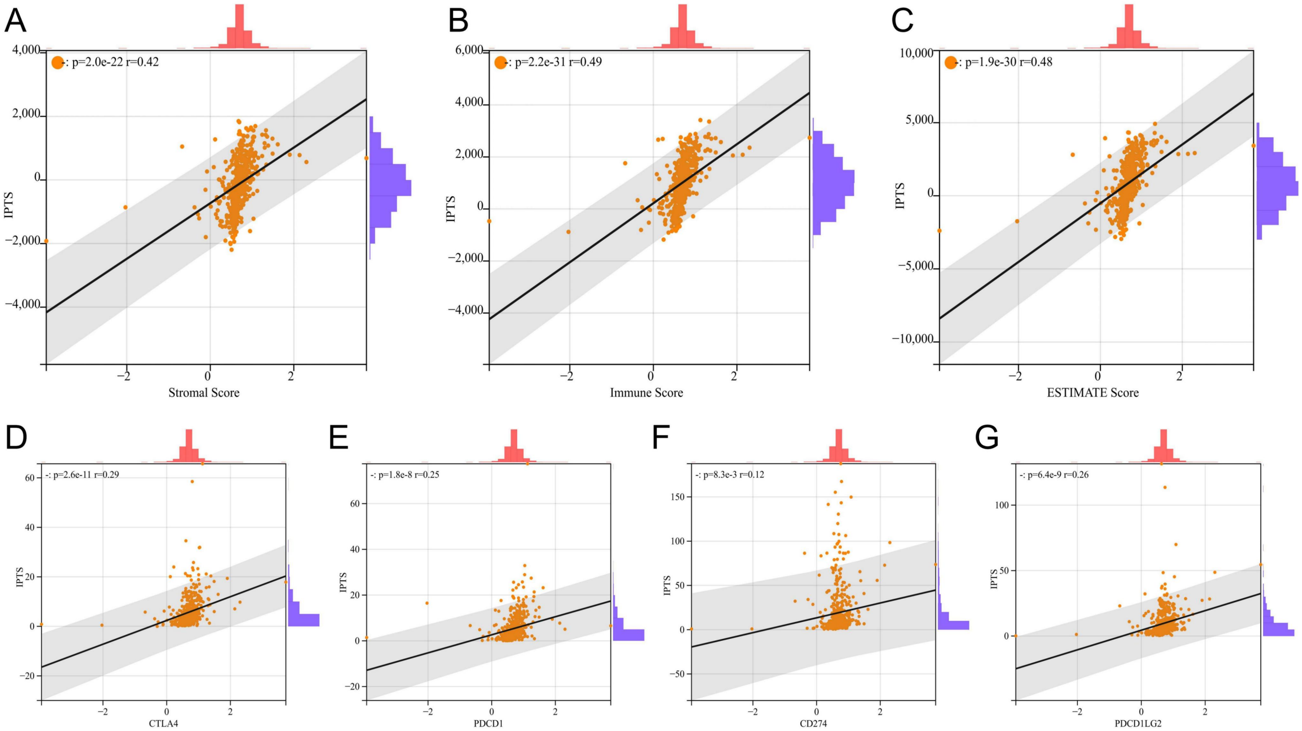

Supplementary Figure 4. Correlation analysis between IPTS and (A) stromal score, (B) immune score, (C) ESTIMATE score, (D) CTLA4 TPM value, (E) PDCD1 (PD-1) TPM value, (F) CD274 (PD-L1) TPM value, and (G) PDCD1LG2 (PD-L2) TPM value.
